# Long-Term Survival of a Cat with Primary Leiomyosarcoma of the Urinary Bladder

**DOI:** 10.3390/vetsci6030060

**Published:** 2019-06-27

**Authors:** Anneliese Baetz Buzatto, Fabiana Elias, Mayara Simão Franzoni, Carlos Eduardo Fonseca-Alves

**Affiliations:** 1 Veterinary Oncology Specialization Course, Instituto Bioethicus, Botucatu SP 18605-545, Brazil; 2 Superintendencia Unidade Hospitalar Veterinaria Universitaria, Federal University of the Fronteira Sul, Realeza PR 85770-000, Brazil; 3 Department of Veterinary Surgery and Anesthesiology, School of Veterinary Medicine and Animal Science, University of Sao Paulo State – UNESP, Botucatu SP 18618-681, Brazil

**Keywords:** leiomyosarcoma, urinary bladder, cat

## Abstract

Primary bladder leiomyosarcoma was diagnosed in a four-year-old, mixed-breed, spayed female cat that presented with lethargy, stranguria, polyuria, hematuria, urinary incontinence and abdominal sensitivity. On abdominal ultrasound, the urinary bladder was observed to have a preserved anatomical position and a hyperechoic mass. The mass measured approximately 1.5 cm, was irregular, and arose from the mucosa of the bladder wall. Due to the evidence of a primary tumor in the urinary bladder, we conducted a partial cystectomy with a 1.0 cm surgical margin and performed histopathology and immunohistochemistry. The histopathology revealed a poorly differentiated malignant neoplasm, characterized by the proliferation of spindle cells with moderate nuclear pleomorphism, suggestive of leiomyosarcoma. Immunohistochemistry confirmed the histopathological diagnosis, showing positive staining for vimentin, desmin and alpha-smooth muscle actin and negative staining for S100, pan-cytokeratin and MyoD1. We also assessed the proliferative index by Ki67 staining and found that 57% of the neoplastic cells were positive for Ki67. We conducted clinical follow-ups every three months in the first year and every six months thereafter. The patient showed no signs of recurrence after 48 months. The surgery was sufficient to treat the leiomyosarcoma, and adjuvant chemotherapy was not necessary in this case.

## 1. Introduction

Feline bladder tumors are very rare in veterinary medicine. Primary transitional cell carcinoma [1,2,3] are the most common tumor subtype. Besides transitional cell carcinoma, previous reports have described a lymphoma [4,5,6], fibrosarcoma [7], and leiomyosarcoma [3,8,9]. The clinical signs are variable, and patients commonly show hematuria, polyuria, dysuria, urinary incontinence, and lower urinary tract infections [1,2,3,4,5,6]. Leiomyosarcomas are mesenchymal malignant tumors of smooth muscle, and there is little information regarding the treatment of leiomyosarcoma in cats.

Due to the limited information regarding the diagnosis and outcomes of urinary leiomyosarcomas in cats, we describe herein a case of primary bladder leiomyosarcoma in a female cat.

## 2. Case Description 

**Figure 1:**
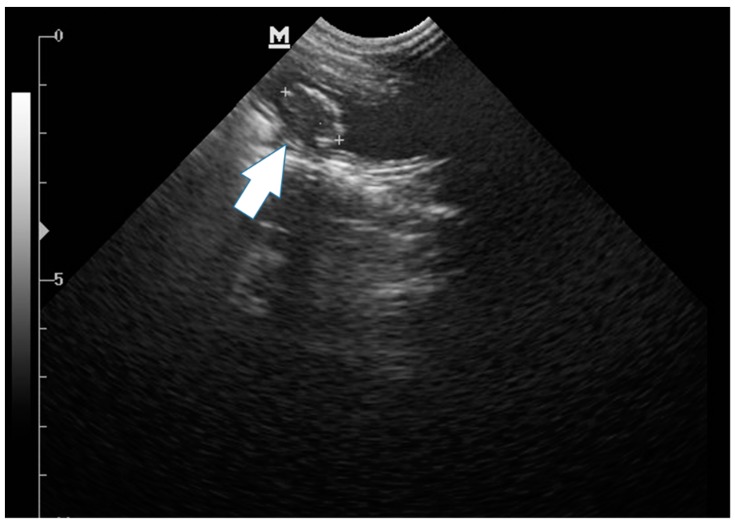
Longitudinal ultrasonographic image of the urinary bladder obtained with a curvilinear transducer of a primary bladder leiomyosarcoma. An ovoid adherent mass (white arrow) is apparent in the bladder lumen. Longitudinal ultrasonographic image of the urinary bladder obtained with a curvilinear transducer.

A four-year-old, mixed-breed, spayed female cat that presented with apathy, stranguria, polyuria, hematuria, urinary incontinence and abdominal sensitivity was referred to the veterinary hospital. The owner described a one-year history of previous treatment with antibiotics and a commercial diet (Royal Canin Urinary^®^, Aimargues, France) for recurrent urinary disorders. However, after cessation of treatment, there was a recurrence of the clinical signs. Due to the history of chronic lower urinary tract disease, a complete blood count (CBC) was performed, a dosage of creatinine was administered, and urea, urinalysis, and uroculture tests were performed with an antibiogram and abdominal ultrasonography. The CBC showed increased hemoglobin (18.0 g/dL), hematocrit (50.0%), eosinophils (2000/µL), lymphopenia (900/µL) and total protein (8.1). There were no alterations in creatinine (1.1 mg/dL) or urea (30 mg/dL) values. Urinalysis revealed hyposthenuria, proteinuria (500 mg/dL), hematuria, and bacteriuria. The uroculture evidenced the growth of Bacillus sp. (>100,000 CFU/mL), which was sensitive to enrofloxacin. Abdominal ultrasonography revealed a poorly filled urinary bladder, with a preserved anatomic shape, showing an irregular mass measuring 1.5 cm in diameter that was adhered to the wall, and exhibited areas of calcification (Figure 1).

**Figure 2:**
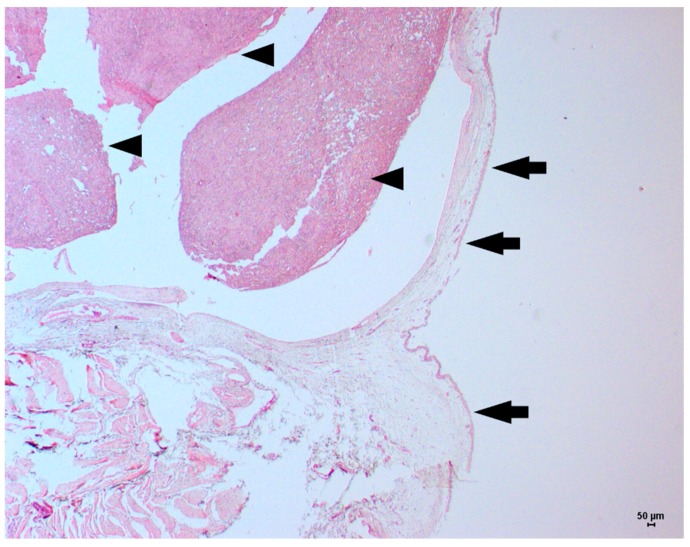
Low-power magnification view of a feline bladder leiomyosarcoma. The intact urothelial epithelium in the bladder mucosa (black arrows) and the fusiform tumor cells growing in the bladder submucosa (arrowhead) can be observed. Hematoxylin and eosin staining, original magnification 2.5×.

**Figure 3:**
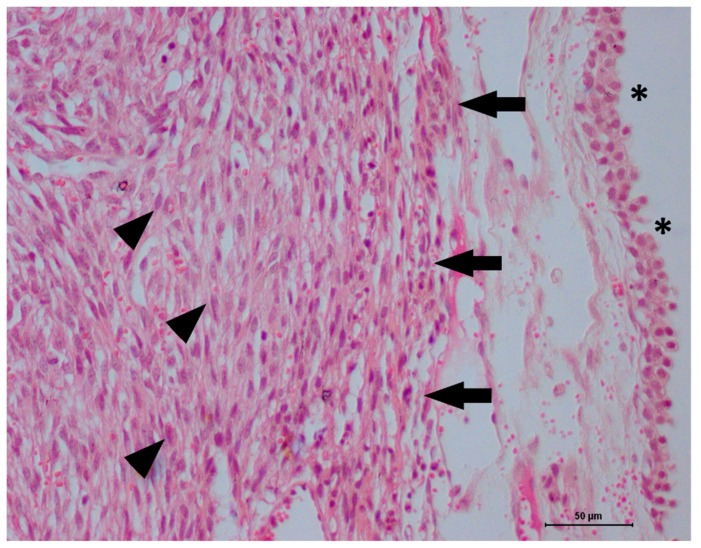
High-power magnification view of a feline bladder leiomyosarcoma. A normal urothelial cell in the bladder mucosa (asterisk), and a mesenchymal tumor in the bladder submucosa (black arrows) can be observed. The tumor cells exhibit a fusiform shape, with moderate anisokaryosis (arrowhead), and discrete hemorrhagic areas. Hematoxylin and eosin staining, original magnification 40×.

Thus, medical treatment with enrofloxacin (5 mg/kg SID for 7 days) and ketoprofen (1 mg/kg SID for 5 days) was performed. Then, a partial cystectomy was performed, with the whole tumor mass removed with a 1.0 cm surgical margin. The gross morphology of the tumor mass revealed a brownish nodule with a lobed and ulcerated surface measuring 2.2 × 1.3 × 0.8 cm. The histological evaluation revealed a poorly differentiated malignant neoplasm ulcerating the bladder mucosa (Figure 2). The neoplasm was characterized by fusocellular proliferation of cells with moderate nuclear pleomorphism, round to oval nuclei, and eosinophilic cytoplasm, interspersed by elongated cells with pale, irregular nuclei. Multiple nucleoli formed bundles and arrays with myxoid areas. The mitotic index was 15, as evaluated in 10 high-power fields. The histopathological evaluation suggested a leiomyosarcoma (Figure 3). Additional diagnostic tests to confirm this diagnosis, including Masson’s trichrome staining and immunohistochemistry for S100, myoblast determination protein 1 (MyoD1), pan-cytokeratin, vimentin, desmin, alpha-actin and Ki67, were performed according to Alves et al. [8].

The immunohistochemical evaluation was performed using citrate buffer (pH 6.0) and a pressure cooker (Pascal®, Dako Cytomation, Carpinteria, CA, USA). The slides were placed in an automatic immunohistochemical processing unit, in an Autostainer Classic® platform (Dako Cytomation, Carpinteria, CA, USA). The antibody information is described in Table 1. A polymer system was used for incubation with a secondary antibody (Envision, Dako Cytomation, Carpinteria, CA, USA) for one hour, and immunoreactions were revealed with 3,3’-diaminobenzidine (DAB). Counterstaining was performed with Harris hematoxylin. Positive and negative controls were prepared according to Alves et al. [10].

**Figure 4:**
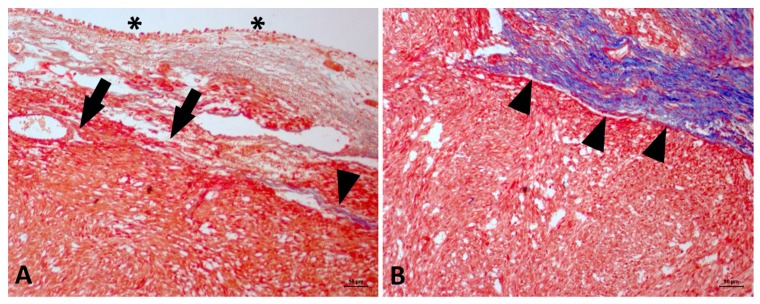
Feline bladder leiomyosarcoma. (**A**) Red staining in normal mucosa (urothelial epithelium, asterisk) and of tumor cells (black arrows) is shown, supporting the leiomyosarcoma diagnosis. The remaining collagen fibers are stained in blue (arrowhead). (**B**) Contrast between the blue staining of the collagen fibers (arrowhead) and the red staining of the tumor cells. Masson’s trichrome stain, original magnification 20×.

**Figure 5:**
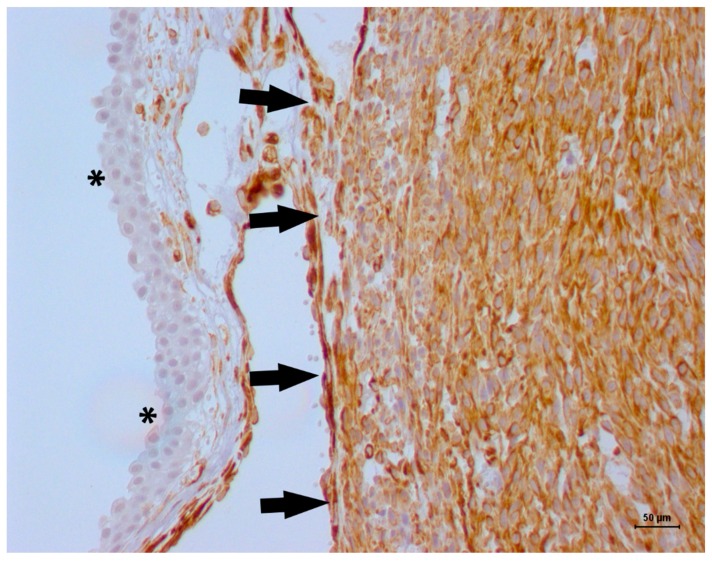
Immunohistochemical evaluation for alpha-actin in a feline bladder leiomyosarcoma. The transitional cell layer of the bladder mucosa was negative for alpha-actin (blue staining, asterisk), and the tumor cells were diffusely positive for alpha-smooth muscle actin (black arrows). Harris hematoxylin counterstaining, DAB, original magnification 20×.

**Figure 6:**
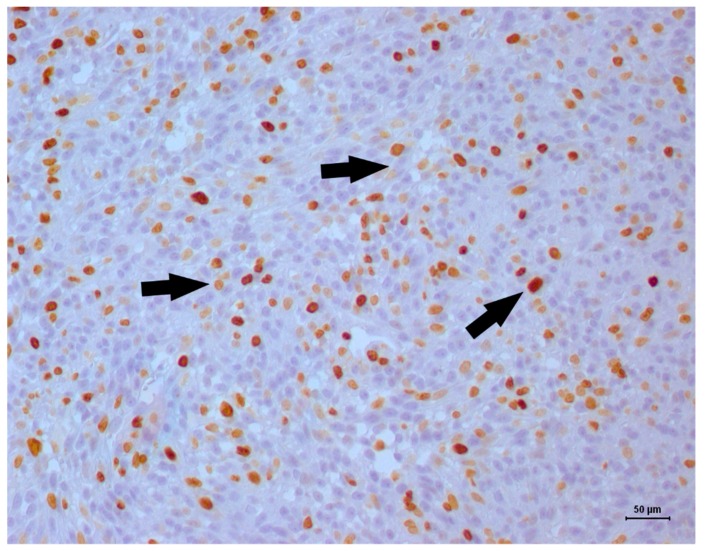
Ki67 immunostaining in a feline bladder leiomyosarcoma. Positive nuclear staining occurred in greater than 50% of the neoplastic cells, which indicated a high proliferative index (black arrows). Harris hematoxylin counterstaining, DAB, original magnification 20×.

The tumor cells stained red with Masson’s trichrome, and only the infiltrating fibroblasts were found in the collagen matrix (stained blue, Figure 4). In the immunohistochemical staining, the tumor cells were negative for pan-cytokeratin, S100 and MyoD1. However, the cells were diffusely positive for vimentin, desmin and alpha-actin (Figure 5). We also observed that 57% of the tumor cells stained positive for Ki67 (Figure 6). The histochemical and immunohistochemical evaluations confirmed the leiomyosarcoma diagnosis.

Since there existed no description of adjuvant therapy for feline leiomyosarcoma in the bladder, adjuvant therapy was not administered, and we performed a clinical follow-up every three months during the first year. From the second year onwards, follow-up was performed every six months. The follow-up exams included abdominal ultrasounds, three-view thoracic X-rays and CBCs. No abnormalities or tumor remission were found in any of the exams during the follow-up evaluations, and after four years of follow-up the patient was considered cured. A follow-up urine culture was not performed, and resolution of clinical sings occurred after seven days post-surgery. 

## 3. Discussion

Feline bladder leiomyosarcomas are extremely rare in veterinary medicine, and there are only three previous reports of these sarcomas in the literature. In the present case, the patient had a history of recurrent feline lower urinary tract disease (FLUTD). The association between FLUTD and bladder neoplasm has been described in the literature [1,2,3,4,5,6,7]. Inflammation is usually associated with transitional cell carcinomas [11]. Inflammatory cells produce various cytokines and enzymes, such as cyclooxygenase-2 (COX-2). The cytokines produced by the inflammatory microenvironment induced cell damage and malignant proliferation [11].

The ultrasound evaluation is useful to identified tumor patterns and infiltration into bladder wall [3,4,5,6]. Usually, lymphomas seem to be more infiltrative than transitional cell carcinoma, or mesenchymal tumors. Thus, for lymphomas, infiltration of the bladder wall is expected, and for carcinomas and sarcomas, a proliferative growth pattern, whether or not associated with bladder wall infiltration, is expected [5,6,7,8,9]. Interestingly, in this case and other reported in the literature [6,7], the affected cats showed the tumor diagnosis at a young age (lower than five years), and polypoid cystitis should be a differential diagnosis [12]. 

Regarding bladder sarcomas, there is little information regarding the association between inflammation and tumor development. Three previous reports in the literature [3,8,9] described the presence of FLUTD and bladder leiomyosarcoma (Table 2). In humans, spindle cell tumors represent important differential diagnoses for undifferentiated bladder tumors. This entity is an epithelial tumor showing undifferentiated cells with a mesenchymal morphology. Our histochemical and immunohistochemical panel was sufficient for a definitive diagnosis. Since the tumor cells were negative for S100 and pan-cytokeratin, we excluded a neuronal or epithelial origin, respectively. The red staining of tumor cells using Masson’s trichrome stain excluded a fibrosarcoma diagnosis.

Vimentin staining indicated a tumor from mesenchymal origin, whereas desmin demonstrated a tumor originating from muscle. The alpha-actin is protein from smooth muscle fibers, and MyoD1 from skeletal muscle fibers [10]. The positive vimentin, desmin, and alpha-actin expression, and negative MyoD1 expression, supported the leiomyosarcoma diagnosis. The Ki67 expression suggested a high proliferative index (57% of tumor cells). There is no information available to date regarding adjuvant therapy in feline bladder leiomyosarcoma. In the two previous reports [6,7], adjuvant therapies were not performed. In the Burk et al. [7] report, tissue biopsy was performed for definitive diagnosis, and after one month euthanasia was performed due to uremia and progressive tumor growth. In the Patnaik and Greene [6] report, a leiomyoma was treated with surgery, with no adjuvant therapy, and the patient achieved 25 months of survival. Since it is a benign tumor, a high survival time, followed by surgery, is expected. Thus, our report describes the highest survival time for a patient with bladder leiomyosarcoma treated with surgery. In this case, surgical approach was sufficient to achieve long-term survival and no adjuvant therapy was required.

There was described in the previous literature, a two bladder fibrosarcoma treated with surgery [12,13]. In one case, a complete surgery was performed; however, the owner declined to perform a complete follow-up and reported absence of clinical signs 16-months after surgery [12]. Since there was an incomplete clinical follow-up, we can infer that surgery was sufficient to achieve a long-term survival. In the other case [13], there was an incomplete surgical removal and, due to the local invasion, euthanasia was performed eight-months after surgery. In our case, we performed a complete imaging fallow-up proving the patient’s disease-free status. Besides that, even treating with surgery without margins, tumor remission was achieved. 

## 4. Conclusions

Even with a high proliferative index, a surgical approach was sufficient to provide long-term survival for the cat studied in this report. Feline leiomyosarcoma should be included in the differential diagnosis of patients with chronic lower urinary tract disease and histochemistry. Immunohistochemistry can be used for the definitive diagnosis. 

## Figures and Tables

**Table 1:** Primary antibodies used for immunohistochemical analysis.

Antibody	Dilution	Clone	Manufacturer
S100	1:400	Polyclonal	Dako Cytomation (Carpinteria, CA, USA)
MyoD1	1:200	5.8A	Novocastra (Wetzlar, Germany)
Pan-cytokeratin	1:300	AE1/AE3	Invitrogen (Carlsbad, CA, USA)
Vimentin	1:300	V3	Invitrogen (Carlsbad, CA, USA)
Desmin	1:100	D33	Dako Cytomation (Carpinteria, CA, USA)
Alpha-actin	1:100	1A4	Dako Cytomation (Carpinteria, CA, USA)
Ki67	01:50	MIB1	Dako Cytomation (Carpinteria, CA, USA)

**Table 2:** Summary of the published cases involving primary bladder leiomyosarcoma in cats.

Reference	Primary Diagnosis	Number of Cases	Treatment	Overall Survival
Patnaik and Greene [8]	Leiomyoma	1	Surgery	25 months
Burk et al. [9]	Leiomyosarcoma	1	Proteolytic enzymes	1 month
Patnaik et al. [3]	Leiomyosarcoma	2	ND	ND

ND: Not described.
